# Save our Sight (SOS): a collective call-to-action for enhanced retinal care across health systems in high income countries

**DOI:** 10.1038/s41433-023-02540-w

**Published:** 2023-06-06

**Authors:** Anat Loewenstein, Alan Berger, Avril Daly, Catherine Creuzot-Garcher, Richard Gale, Federico Ricci, Javier Zarranz-Ventura, Robyn Guymer

**Affiliations:** 1 Ophthalmology Division, Tel Aviv Medical Center, Tel Aviv University, Tel Aviv, Israel; 2 St. Michael’s Hospital, University of Toronto, Toronto, ON Canada; 3 Toronto Retina Institute, Toronto, ON Canada; 4 Retina International, Dublin, Ireland; 5 Dijon University Hospital, Dijon, France; 6 Hull York Medical School, University of York, York, UK; 7 York and Scarborough Teaching Hospitals NHS Foundation Trust, York, UK; 8 Dept. Experimental Medicine - University Tor Vergata of Rome, Rome, Italy; 9 Hospital Clinic of Barcelona, University of Barcelona, Barcelona, Spain; 10 August Pi and Sunyer Biomedical Research Institute, University of Barcelona, Barcelona, Spain; 11 Centre for Eye Research, Royal Victorian Eye and Ear Hospital, University of Melbourne, Melbourne, VIC Australia

**Keywords:** Quality of life, Health services, Retinal diseases, Diagnosis, Therapeutics

## Abstract

With a growing aging population, the prevalence of age-related eye disease and associated eye care is expected to increase. The anticipated growth in demand, coupled with recent medical advances that have transformed eye care for people living with retinal diseases, particularly neovascular age-related macular degeneration (nAMD) and diabetic eye disease, has presented an opportunity for health systems to proactively manage the expected burden of these diseases. To do so, we must take collective action to address existing and anticipated capacity limitations by designing and implementing sustainable strategies that enable health systems to provide an optimal standard of care. Sufficient capacity will enable us to streamline and personalize the patient experience, reduce treatment burden, enable more equitable access to care and ensure optimal health outcomes. Through a multi-modal approach that gathered unbiased perspectives from clinical experts and patient advocates from eight high-income countries, substantiated perspectives with evidence from the published literature and validated findings with the broader eye care community, we have exposed capacity challenges that are motivating the community to take action and advocate for change. Herein, we propose a collective call-to-action for the future management of retinal diseases and potential strategies to achieve better health outcomes for individuals at-risk of, or living with, retinal disease.

## Abstract (zh)

随着人口老龄化的增长, 年龄相关眼病的患病率和相关眼部护理的需求预计将会增加。预期的需求增长, 加上近期的医疗的进步, 转变了视网膜疾病尤其是新生血管性年龄相关性黄斑变性(nAMD)和糖尿病相关眼病患者的眼部护理, 这为医疗系统主动管理这些疾病的预期负担提供了机会。为此, 我们必须采取集体行动, 通过设计和实施可持续发展战略来解决现有和预期的能力限制, 使医疗系统提供一个最优的护理标准。充足的能力将使我们简化和个性化患者的体验、减轻医疗负担, 有公平的途径来关怀和确保最优的健康结果。通过一种多模式的渠道收集了来自八个高收入国家中的临床专家和患者权益倡导者公正的观点、从已发表的文献中获得论证, 眼科护理界验证了结果, 我们揭示了激励社区采取行动并提倡改革的挑战能力。在此, 我们提出一个集体呼吁活动, 呼吁未来对视网膜疾病进行管理,并为有患病风险或正患有视网膜疾病的人们实现更好的医疗健康结果提出潜在策略。

## Introduction

Vision impairment and blindness are significant public health issues affecting a large proportion of the global population [1]. According to the World Health Organization, more than 2 billion people live with some form of vision impairment or blindness [2]. In addition to the impact on a person’s health and quality of life, vision impairment has an enormous socioeconomic burden, with estimated annual global productivity losses of nearly US$ 411 billion [3]. In the United Kingdom alone, vision impairment results in ~US$ 3.6 billion in direct health system costs annually [4].

Age-related macular degeneration (AMD) and diabetic retinopathy (DR), contribute significantly to the prevalence of vision impairment. Currently, ~196 million people are living with AMD, which is expected to increase to 288 million by 2040 [5]. Similarly, the number of people with diabetes is expected to grow from 463 million to 700 million, and ~22% are expected to develop DR [6, 7]. The rising prevalence of these diseases due to an increasingly aging population is placing significant strain on already overburdened health systems. People living with retinal disease face numerous challenges in receiving timely diagnosis and treatment, navigating complex referral systems and coordinating among healthcare teams. A constrained clinical workforce, inefficient workflows, burdensome treatment options and gaps across the disease management continuum (e.g. screening) are impacting the ability to receive timely and personalized care [1, 8, 9].

Innovations in care and management—from new anti-VEGF therapies to the use of OCT technology—are enhancing our ability to treat individuals with retinal disease, but they will also likely further amplify demand [10, 11]. To alleviate the health, social and economic burden of these diseases, it is imperative for us to proactively identify and advocate for sustainable, tangible strategies to alleviate existing and emerging health system constraints that impede our ability to provide optimal retinal care.

As leaders in the field, we see an opportunity to pave a new path forward for retinal care that will alleviate the burden of disease, improve quality of life and help people living with retinal disease unlock their full potential. We envision a world where *every person at-risk of or diagnosed with retinal disease has timely access to integrated and personalized care in order to achieve their optimal visual outcome and quality of life.* We call on health systems, policymakers and the broader eye care community to work towards trying to achieve the following four goals:

1. Enhance detection and diagnostic capacity to enable early intervention
2. Alleviate treatment burden on patients and health systems
3. Enable better monitoring and management of individuals with retinal disease
4. Improve coordination of care across the patient journey

**Fig. 1:**
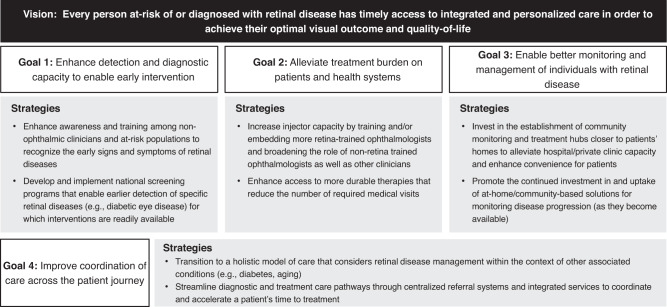
Model for the future of retinal disease care. Schematic representation of our shared vision for the future management of retinal diseases, as well as the four overarching goals and associated strategies to enable progress in achieving our vision.

Below, we offer rationale for the importance of each of these goals, propose potential strategies (or solutions) to help achieve these goals and offer considerations for implementation (Fig. 1; Table 1). Of note, the proposed strategies represent expert perspectives of a highly regarded group of international eye care clinicians and patient advocates—all of whom have first-hand experience with vision health and navigating health systems (see Box 1: Overview of Our Approach for additional details). Given that the experts come from high income countries (HICs), the perspectives and suggestions herein may have greater relevance to these regions as opposed to others (e.g., low- and middle-income countries). It is also important to recognize that while some strategies are more tangible and could represent near-term opportunities, others are more aspirational in nature and may have a longer runway for consideration and implementation.

**Table 1:** Implementation considerations by strategy.

BOX 1 Overview of our approach For this expert consensus paper, we implemented a modified Delphi approach to explore unbiased viewpoints from an international group of clinical experts and patient advocates from HICs—Australia, Canada, France, Ireland, Israel, Italy, Spain, and the United Kingdom—whose health systems face significant pressures and experience similar capacity challenges related to increased demand for eye care. We first conducted one-on-one interviews with each expert to gather perspectives on current and emerging health-system capacity challenges, define a collective vision for the future of retinal care and identify potential strategies to achieve this vision. In parallel, we reviewed the published literature to substantiate (or counter) individual views and perspectives. Key findings were presented and validated first with experts and then discussed as part of broader engagement with the eye care community (11 additional participants, including ophthalmologists, optometrists, nurse consultants and representatives from patient advocacy groups from Israel, Sweden, United Kingdom, Argentina, Canada, Switzerland and Australia). The workshop resulted in additional refinement and validation, allowing regional nuances and perspectives to be integrated into the proposed strategies and implementation considerations.

## Model for future of retinal care

### Goal 1: Enhance detection and diagnostic capacity to enable early intervention

With the rising epidemiological burden of AMD and DR, health systems must be prepared to detect and diagnose retinal diseases as early as possible to mitigate the risk of vision loss [5, 7]. Detection and diagnosis are often challenged by a lack of awareness among the general population of the signs and potential risks of retinal diseases. For example, among nAMD patients who delayed visiting a healthcare provider for changes in their vision, 41% believed that these changes would resolve on their own and 20% assumed these changes were a normal part of aging [12]. The longer retinal diseases go undiagnosed, the more challenging treatment and disease management becomes, often resulting in poorer final visual outcomes. As a result, early detection and diagnosis can afford people with retinal disease more timely access to vision-saving interventions and, ultimately, mitigate the broader social and economic impacts related to the onset of vision impairment and blindness.

To create a system that supports early detection and diagnosis of retinal diseases, we could consider: (i) enhancing awareness and training among non-ophthalmic clinicians and at-risk populations to recognize the early signs and symptoms of retinal diseases; and (ii) developing and implementing national screening programs that enable early detection of specific retinal diseases (e.g. diabetic eye disease) for which interventions are readily available.

#### We encourage enhanced awareness and training among non-ophthalmic clinicians and at-risk populations to recognize the early signs and symptoms of retinal diseases

There is an opportunity for non-ophthalmic clinicians (e.g., GPs, pharmacists, nurses) to assume a larger role in the detection and diagnosis of eye diseases that complements the roles of optometrists and non-retina trained ophthalmologists, including promoting good eye health, visits to eye specialists and supporting early diagnosis and referrals. For non-ophthalmic clinicians at the frontline of care, knowledge and awareness around vision health is often lacking, necessitating training to support a more active role in disease awareness-building and detection [8]. This may occur through medical school training, continuing medical education and/or greater collaboration and coordination across specialties to share knowledge around retinal diseases. At minimum, primary care physicians, nurses, pharmacists and other specialists should be aware of the early warning signs and symptoms of retinal diseases to educate and encourage at-risk patients (e.g., diabetic patients) to have their eyes evaluated regularly to enable earlier diagnosis and support better vision health outcomes.

An expanded role in retinal disease detection for non-ophthalmic clinicians has been explored in some countries, with promising results. In the United States, general practitioners (GPs) trained in reading retinal photographs have been shown to identify nearly 90% of DR referrals (compared to retina-trained ophthalmologists) [13]. To increase DR screening and detection, particularly in rural and remote Indigenous communities, Australia has also invested in training primary care staff and offers Medicare rebates to GPs and endocrinologists for the appropriate use of retinal fundus cameras for DR screening [14]. AI-enabled algorithms—which are used in the United States and have been shown to effectively interpret retinal photographs—could serve as a useful screening aide for non-ophthalmic clinicians and other eye specialists to support detection and diagnosis [15–18].

#### We encourage the development and implementation of national screening programs that enable early detection of specific retinal diseases (e.g., diabetic eye disease) for which interventions are readily available

National screening programs can serve as effective tools for early intervention in retinal diseases where treatment is readily available (e.g., diabetic eye disease), delaying and/or preventing permanent vision loss and shifting the pendulum from reactive to proactive retinal disease management. The UK’s DR screening program (annual eye screening for anyone 12+ with diabetes) is a prime example of the health impact of retina screening programs. Implemented in 2003, DR was no longer the leading cause of blindness among working age adults by 2010. In 2018 alone, 2.23 million people were screened (82.7% of the eligible population), leading to 8782 urgent referrals and 54,893 routine referrals to ophthalmology departments [19]. Further, studies from Spain, Canada and Singapore have found various DR screening strategies to be cost-effective [20–22]. Increasing screening intervals and/or separating patients into high- and low-risk groups may further increase the cost-effectiveness of DR screening [23–25]. As screening programs evolve and become more prominent, the integration of AI algorithms in the screening process could also be explored [15]. The sensitivity in detecting referable DR using an AI-enabled diagnostic model has been shown to be comparable to that of trained graders (90.5% vs. 91.2%), with a slightly lower degree of specificity (91.1% vs. 99.6%) [17]. Additionally, as new treatment options and innovations become available, it may be prudent to explore screening strategies for other retinal diseases (e.g., selected nAMD screening for high-risk individuals) that would identify individuals early in their disease progression. Support for these screening programs would require evidence on feasibility and cost-effectiveness.

### Goal 2: Alleviate treatment burden on patients and health systems

The rapid rise in demand for anti-VEGF therapies has not been matched by a similar increase in injection capacity. Between 2009 and 2019, a single tertiary care hospital in the UK saw the number of annual anti-VEGF injections administered increase nearly 11-fold from 4143 to 44,924 and is forecasted to double again by the end of the decade [26]. This increased demand for retinal care, is contributing to delays in initiating and/or providing treatment and forcing the prioritization of patients based on disease severity or treatment urgency to manage workload (e.g. those with AMD prioritized over DR). From a patient perspective, chronic treatment requiring multiple visits presents a challenge to maintaining adherence to treatment schedules, as do other factors, such as competing health issues and lack of access to caregivers [12, 27]. Ultimately, high treatment burden may lead to under treatment or treatment discontinuation, which is frequently associated with poorer vision outcomes [28]. Similarly, a South Korean study demonstrated that the proportion of eyes with 20/200 or better visual acuity markedly decreased from 71.4% to 17.1% in the two years following treatment discontinuation [29]. To accommodate the treatment burden on patients and individual health systems, we could consider: (i) increasing injector capacity by training and/or embedding more retina-trained ophthalmologists and broadening the role of non-retina trained ophthalmologists as well as other clinicians; and (ii) enhancing access to more durable therapies that reduce the number of required medical visits.

#### We encourage increasing injector capacity by training and/or embedding more retina-trained ophthalmologists and broadening the role of non-retina trained ophthalmologists as well as other clinicians

The sheer volume of injections impedes the ability of retina-trained ophthalmologists to provide personalized care by taking away from potential time spent on other elements of retinal disease management (e.g., meeting with patients, developing treatment plans, interpreting OCT scans etc.). Several potential approaches exist to alleviate this challenge.

One strategy could be to train more ophthalmologists early in their careers to provide injections to treat retinal diseases, or later in their careers through continuing medical education which, would require investment from medical colleges and speciality education programs. In countries such as Italy, there may be an opportunity to explore increasing the number of injectors by refining the way in which injections are given. Currently, injections are required to be performed in surgical theatres in some countries, which limits the number of injections per session and the number of ophthalmologists who can attend the operating suites to give injections. We encourage such regions to reconsider policies that limit where injections can take place, allowing for more widespread treatments (e.g., office-based injections in Canada or dedicated clean injection rooms in the UK) [30–32]. There are many specialists available that could be recruited to enhance injection capacity and improve performance if the enabling environment were more flexible to the location of the injections. Some countries have also adopted the practice of training non-specialists to deliver injections, alleviating the burden on ophthalmologists and allowing them to focus on overall disease management. In Israel, ophthalmology residents can administer anti-VEGF injections, and some European countries have started training and empowering allied healthcare professionals (e.g., ophthalmic nurse practitioners) to deliver anti-VEGF agents. In Denmark, resident physicians and nurses have been shown to successfully administer intravitreal injections without increased risk of complications [33]. In the UK, the Royal College of Ophthalmologists determined that certain non-medical health professionals could administer anti-VEGF agents assuming specific conditions were met, including: (1) an appropriate level of training has been provided; (2) an ophthalmic specialist is available for advice and/or management of complications; (3) processes are in place to enable continuous audit and patient feedback; and, (4) appropriate indemnity and patient consent policies are in place [34]. Such strategies for increasing injector capacity have yielded favorable outcomes, including in Norway where nurse-led injections have been shown to be non-inferior to physician injections with no increased risk to visual function [35]. Further, patients have shown comfort with these injectors; in the UK, 81% of patients had no initial reservations about receiving nurse-led injections and 100% of patients were satisfied with the service they received from a nurse [34]. While this solution has the potential to increase injector capacity, consultation and buy-in from local medical councils would be required to broaden/adjust health professional roles and share responsibilities across clinicians.

#### We encourage enhancing access to more durable therapies that reduce the number of required medical visits

The high volume of injections associated with current treatment regimens is burdensome to both clinicians and patients. While durable treatment options do exist and treatment paradigms intended to reduce unnecessary visits have been implemented (e.g., treat and extend), we want to do better. Health systems must ensure broad adoption of durable therapies to alleviate treatment burden and provide each person living with retinal disease access to the innovations with the best chance of improving outcomes. Across HICs, the cost of more durable treatments is often a limiting factor for reimbursement, resulting in limited access to certain therapies and/or a focus on less expensive, short-duration drugs. Mandates for shorter duration first-line therapies and restrictions on the use of more durable therapies also limit access (as seen in Canada and Italy). Shifting the narrative towards the full value of durable, more efficacious therapies will be paramount to enhancing the patient experience and improving overall health and vision outcomes.

In addition, investment in research and innovation will be critical to bring the next generation of durable therapies and innovative solutions to patients. Indeed, it seems likely that more durable treatment options, with the potential to extend injection intervals to four months, or longer—as shown with implantable devices that deliver ongoing anti-VEGF treatment—will become commercially available in the near future [36–39]. Less invasive interventions, including oral medications and subcutaneous injections, are also being explored to mitigate the need for intravitreal injections [40, 41]. Additionally, research into new mechanisms of action and additional disease pathways beyond anti-VEGFs are being explored, which may further improve treatment durability in the future. Treatment options that reduce the burden on clinicians and patients while retaining high efficacy and safety will become valuable to managing the burden of retinal diseases in the future.

### Goal 3: Enable better monitoring and management of individuals with retinal disease

Individuals with retinal disease often experience logistical challenges related to attending their appointments, such as distance, time and costs. As reported in France, distance to treatment sites (51.7%) and burden of periodic follow-up visits (24.1%) were among the top reasons for treatment discontinuation in AMD patients [11]. Further, many individuals with retinal disease depend on caregivers, who bear a significant burden on their productivity and quality of life. An Australian study estimated that caregivers spend 6.2 h per month accompanying AMD patients to their appointments, equating to monthly losses of AUD $262.45 in income based on the national hourly wage [42]. While customized protocols (e.g., treat-and-extend) and more durable therapies allow for more tailored care, the influx of new patients requiring chronic treatment/monitoring is exceeding the number of patients able to be treated, making it difficult to meet patient and disease management needs.

To enable better monitoring and management of individuals with retinal disease, we could consider: (i) investing in the establishment of community monitoring and treatment hubs closer to patients’ homes to alleviate hospital/private clinic capacity and enhance convenience for patients; and (ii) promoting the continued investment in and uptake of at-home/community-based solutions for monitoring disease progression (as they become available).

#### We encourage investing in the establishment of community monitoring and treatment hubs closer to patients’ home to alleviate hospital/private clinic capacity and enhance convenience for patients

Community monitoring hubs could offer patients access to disease monitoring support within primary and secondary care settings (e.g., community hospitals, pharmacies, primary care settings) and with non-specialists (e.g., optometrists). A pilot study in the UK evaluated a community-based model whereby optometrists completed a series of online lectures and case-based learnings to receive accreditation for the management of secondary care quiescent nAMD patients. Following completion of this process, 89% of community optometrists felt confident (58%) or very confident (31%) in monitoring AMD progression; this practice was well accepted by both patients and practitioners [43]. Additionally, an optometrist-led clinic for stable AMD patients in the UK was successful in recommending follow-up plans that aligned with ophthalmologist recommendations [44]. Investing in and continuing to pilot a community-based approach to monitoring retinal disease progression could have benefits to easing the logistical burden on patients and caregivers, freeing up system capacity and bringing care closer to the patient’s home.

#### We encourage promoting the continued investment and uptake of at-home/community-based solutions for monitoring disease progression (as they become available)

Home monitoring technologies could allow for more convenient and real-time assessment of disease progression, in addition to encouraging continued—but less demanding—engagement between the patient and provider. While technologies monitoring *disease progression* are still in their infancy, products that monitor *conversion* to wet AMD are beginning to be adopted around the world. For instance, one technology has been shown to successfully detect conversion from intermediate to wet AMD and has demonstrated high usability among older adults. In real-world settings, 69% of 306 cases with confirmed disease conversion to wet AMD were identified by a system alert prior to a routine or symptom-driven visit [45]. This monitoring system has also been shown to be cost-effective in monitoring AMD patients at risk for choroidal neovascularization compared to scheduled appointments alone [46]. An opportunity exists to broaden the uptake of devices similar to this one to enable at-home monitoring of disease conversion for AMD, thereby empowering patients to monitor their vision health. Further, as evidence for and support of home monitoring technologies grows, investment in and the development of technologies that monitor disease progression may offer a valuable tool to support retinal disease management.

### Goal 4: Improve coordination across the patient journey

Managing multiple conditions can make patient engagement with the health system daunting and complex. Indeed, it has been shown that >50% of people living with diabetic macular oedema average ~19 appointments (totaling 20 h) across multiple care providers over a six-month period [47]. While co-morbidities and risks associated with retinal diseases are well-known, patient care is often fragmented and not coordinated among specialists, resulting in patients being overwhelmed by the volume and frequency of medical appointments. Additionally, the lack of well-designed and efficient referral systems as well as insufficient diagnosis and treatment infrastructure relative to demand is considered a major challenge for retinal disease management and a significant burden to clinicians, patients and health systems around the world.

To enable greater coordination of care along the patient journey, we could consider (i) transitioning to a holistic model of care that considers retinal disease management within the context of other associated conditions (e.g., diabetes, aging); and (ii) streamlining diagnostic and treatment care pathways through centralized referral systems and integrated services to coordinate and accelerate a patient’s time to treatment.

#### We encourage the transition to a holistic model of care that considers retinal disease management within the context of other associated conditions (e.g., diabetes, aging)

In individuals with diabetes, observed changes in the eye may signal potential issues with other organ systems (e.g., kidneys, heart) and could have implications for how individuals manage other aspects of their disease (e.g., reading a glucose monitor). However, this information is not always communicated between healthcare providers. Similarly, older individuals are often managing multiple co-morbidities, including age-related eye diseases (i.e., AMD), and would greatly benefit from a more coordinated approach to care. Transitioning to more holistic and personalized models of care that consider retinal diseases within the context of other associated conditions would create a more seamless patient experience (i.e., reducing travel requirements, simplifying appointments). For example, the Cardiac and Renal Endocrine Clinic at Toronto General Hospital in Canada is a once-monthly multidisciplinary clinic for diabetic patients with multiple co-morbidities. The clinic allows separate disciplines, including ophthalmology, to consult with patients on the same appointment day and develop a single care management plan [48]. Greater adoption and expansion of such models of care will further enable patient-centered care and ensure that vision health is not deprioritized or overshadowed for patients with complex conditions.

#### We encourage streamlining diagnostic and treatment care pathways through centralized referral systems and integrated services to coordinate and accelerate a patient’s time to treatment

Many health systems rely on non-centralized referral systems that make it challenging to triage patients and exchange information between primary care clinicians and specialists, leading to increased wait times, appointment redundancies and treatment delays. Several strategies can be considered to create more efficient care pathways for patients. Centralized referral systems can help improve triaging and decrease referral times, especially for high-risk patients. Australia and Canada (Alberta) have introduced electronic referral systems that have been well-received by patients and clinicians, though factors such the inability to share patient images (e.g., OCT) and subscription costs have limited broader adoption. In 2010, the Scottish government launched the *Scottish Eyecare Integration Project* to develop e-referral infrastructure that enables direct electronic referrals with digital images between community optometrists and hospital eye services [49]. In pilot studies, the introduction of electronic referral pathways was determined to be safe and feasible and shown to reduce unnecessary referrals by 37% compared to traditional paper methods [49, 50]. By 2020, the Scottish National Eyecare Service launched a national, open-source electronic patient record system that allows eyecare records to be shared between primary and secondary care clinicians across the country, allowing for digitized workflows, reduced wait times and hospital visits, and better coordination between clinicians [51]. Fast-track referral processes present another near-term strategy. The UK has implemented a fast-track referral program for urgent referrals of patients with nAMD, retinal vein occlusion and other sight-threatening conditions directly from an optometrist or GP to a specialist triage center [52, 53]. Additionally, one-stop clinics (e.g., regional macular centers) where diagnosis and treatment are provided on the same day have been implemented in the UK, Spain and Italy and are becoming increasingly common around the world. Real-world evidence from the UK found that one-stop clinics used less staff resources and had a shorter appointment duration (4.6 h) compared to the cumulative time spent in two-stop injection and monitoring clinics (7.6 h in total), a benefit to both patients, caregivers and providers [54]. Reflecting on the pilot programs implemented around the world, it is evident that accelerated diagnostic and treatment care pathways will streamline the patient experience, create a more efficient health system and enhance system capacity.

## Implementation considerations

**Fig. 2:**
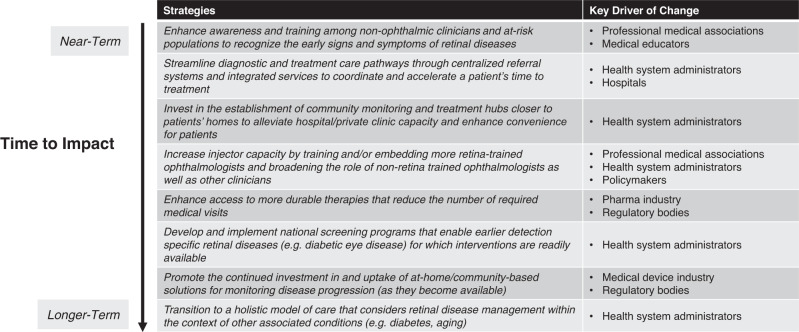
Time to impact across strategies. Relative time to impact for each strategy that considers the degree of system-level change, health system engagement and investment needed for success.

Our consensus paper has put forward a shared vision and goals for retinal disease management and defined potential strategies that will help make this vision a reality. Delivering on these strategies will require careful planning and coordination, as well as engagement with policymakers, health system decision-makers and other stakeholders. We appreciate the lens through which our goals and potential strategies have originated—reflecting nuanced perspectives from HICs. Herein, we have proposed both tangible (near-term) and aspirational (long-term) strategies, describing these as options to consider rather than a fully prescribed roadmap. Ultimately, this paper is intended to be foundational in nature and inspire future conversations and efforts at the level of individual countries or regions to prioritize, test and implement strategies that can achieve our vision for retinal disease management. To support initial discussions, we have outlined implementation considerations for each strategy (Table 1). We have also offered perspectives on the time to impact for each strategy that reflects the degree of system-level change, health system engagement and investment needed for success (Fig. 2).

## Conclusion and future directions

The time is now for us to collectively reimagine what the standard of care for retinal diseases should be and how we can accelerate change in our health systems to meet the needs of individuals living with retinal disease—today and in the future. We can no longer afford to operate as status quo while the demand for retinal care continues to grow exponentially. We encourage regional advocacy partners, policymakers and other eye care leaders to work together in furthering these discussions and bringing the eye care community closer to realizing our collective vision and goals. Further discussion is needed to understand the unique retinal care challenges experienced in low- and middle-income countries and determine if and how the strategies we’ve proposed can apply to these contexts. Together, we can enact meaningful change for health systems to prioritize retinal care and strive towards securing an optimal quality of life for all individuals at risk of or living with retinal disease.

## Summary

### What was known before

- The rising prevalence of age-related retinal diseases is placing significant strain on already overburdened health system capacity.
- People living with retinal disease face numerous challenges in receiving timely diagnosis and treatment, navigating complex referral systems, and coordinating across healthcare teams.
- A constrained clinical workforce, inefficient workflows, burdensome treatment options, and gaps across the disease management continuum are impacting the ability to receive timely and personalized care.
- To alleviate the health, social and economic burden of these diseases, it is imperative for us to proactively identify and advocate for sustainable, tangible strategies to alleviate existing and emerging health system constraints that impede our ability to provide optimal retinal care.

### What this study adds

- A shared vision for the future management of age-related retinal diseases based on perspectives from an international group of eye care clinicians and patient advocates with first-hand experience with vision health and navigating health systems.
- Four overarching goals to achieve this shared vision for retinal disease management: (1) enhancing detection and diagnostic capacity to enable early intervention; (2) alleviating treatment burden on patients and health systems; (3) enabling better monitoring and management of individuals with retinal disease; and (4) improving coordination of care across the patient journey.
- Eight proposed strategies or solutions designed to achieve the defined goals, as well as insights into potential implementation considerations (e.g. general, policy or research-related).
- A collective call-to-action for health systems, policymakers and the broader eyecare community to improve the quality of retinal care now and in the future.
