# Correction: Save our Sight (SOS): a collective call-to-action for enhanced retinal care across health systems in high income countries

**DOI:** 10.1038/s41433-023-02662-1

**Published:** 2023-08-01

**Authors:** Anat Loewenstein, Alan Berger, Avril Daly, Catherine Creuzot-Garcher, Richard Gale, Federico Ricci, Javier Zarranz-Ventura, Robyn Guymer

**Affiliations:** 1 Ophthalmology Division, Tel Aviv Medical Center, Tel Aviv University, Tel Aviv, Israel; 2 St. Michael’s Hospital, University of Toronto, Toronto, ON Canada; 3 Toronto Retina Institute, Toronto, ON Canada; 4 Retina International, Dublin, Ireland; 5 Dijon University Hospital, Dijon, France; 6 Hull York Medical School, University of York, York, UK; 7 York and Scarborough Teaching Hospitals NHS Foundation Trust, York, UK; 8 Department of Experimental Medicine, University Tor Vergata of Rome, Rome, Italy; 9 Hospital Clinic of Barcelona, University of Barcelona, Barcelona, Spain; 10 August Pi and Sunyer Biomedical Research Institute, University of Barcelona, Barcelona, Spain; 11 Centre for Eye Research, Royal Victorian Eye and Ear Hospital, University of Melbourne, Melbourne, VIC Australia

**Keywords:** Quality of life, Health services, Retinal diseases, Diagnosis, Therapeutics

Correction to: *Eye* 10.1038/s41433-023-02540-w, published online 6 June 2023

The article “Save our Sight (SOS): a collective call-to-action for enhanced retinal care across health systems in high income countries”, written by Anat Loewenstein, Alan Berger, Avril Daly, Catherine Creuzot-Garcher, Richard Gale, Federico Ricci, Javier Zarranz-Ventura and Robyn Guymer was originally published electronically on the publisher’s internet portal on 6 June without open access. With the author(s)’ decision to opt for Open Choice the copyright of the article changed on 17 June 2023 to © The Authors 2023 and the article is forthwith distributed under a Creative Commons Attribution 4.0 International License, which permits use, sharing, adaptation, distribution and reproduction in any medium or format, as long as you give appropriate credit to the original author(s) and the source, provide a link to the Creative Commons licence, and indicate if changes were made. The images or other third party material in this article are included in the article’s Creative Commons licence, unless indicated otherwise in a credit line to the material. If material is not included in the article’s Creative Commons licence and your intended use is not permitted by statutory regulation or exceeds the permitted use, you will need to obtain permission directly from the copyright holder. To view a copy of this licence, visit http://creativecommons.org/licenses/by/4.0/. The original article has been corrected.

